# The Role of Cognitive Emotion Regulation Strategies on Problematic Smartphone Use: Comparison between Problematic and Non-Problematic Adolescent Users

**DOI:** 10.3390/ijerph16173142

**Published:** 2019-08-28

**Authors:** Natalio Extremera, Cirenia Quintana-Orts, Nicolás Sánchez-Álvarez, Lourdes Rey

**Affiliations:** 1 Faculty of Psychology, University of Málaga, Campus de Teatinos s/n, 29071 Málaga, Spain; 2 Department of Social, Evolutionary and Educational Psychology, University of Huelva, Faculty of Educational Sciences, Av. Fuerzas Armadas, 21007 Huelva, Spain

**Keywords:** cognitive emotion regulation, problematic smartphone use, coping profile, mobile phone usage, adolescence

## Abstract

Prior work has suggested that individuals with deficits in emotion regulation skills are prone to compulsive behaviour and to following maladaptive coping strategies, such as smartphone overuse, to manage negative moods. Adolescence is a vulnerable developmental stage for deficits in emotion regulation, and these are linked to excessive smartphone use. The present study is the first to examine the links between the use of specific cognitive emotion regulation (CER) strategies and problematic smartphone use in a sample of adolescents. A total of 845 Spanish adolescents (455 females) completed the Spanish versions of the Cognitive Emotion Regulation Questionnaire and the Smartphone Addiction Scale, along with a socio-demographic survey. The adolescents were divided into two groups: Non-problematic smartphone users (*n* = 491, 58.1%) and problematic smartphone users (*n* = 354, 41.9%). Significant group differences were found, with the problematic users reporting significantly higher scores for all maladaptive CER strategies, including higher self-blame, rumination, blaming of others and catastrophising. The results from logistic regression analyses show that rumination, catastrophising and blaming of others were the most important variables for distinguishing between the two groups, along with gender and parental control outside the home. In summary, these findings suggest the importance of specific maladaptive CER strategies in problematic smartphone use and provide insight for relevant targets for intervention designs.

## 1. Introduction

Among other electronic devices and communication technologies, the smartphone has attracted millions of users worldwide, in part because of the wide range of services and functions provided [1]. Indeed, smartphones are carried everyday wherever people go, along with their keys and money [2]. Despite the benefits, there is a dark side to smartphone usage, relating to adverse and negative psychological consequences [3]. Some people may be involved in the excessive use of smartphones, losing control and, in some cases, engaging in an addiction-like disorder [4] that exacerbates personal or social problems [5]. According to Billieux and colleagues [6,7], problematic smartphone use can be defined as a heterogeneous and multidimensional phenomenon involving a pattern of dependency related to negative consequences (e.g., difficulty in focusing on daily activities or on an interpersonal encounter due to a constant need to check smartphone notifications) [8]. It is argued that excessive or problematic smartphone use shares characteristics with other addictive behaviours, including craving, tolerance, withdrawal and mood modification [8].

The prevalence of problematic smartphone use varies between populations and measures, reporting ranges between 0% and 38% [9]. In Spain, 100% of adolescents aged 14–19 years use the smartphone as their favourite device for any activity [10]. Moreover, the majority of Spanish adolescents possess their own smartphone (e.g., 94.8% of 15-year-olds have their own smartphone) [11] and around 20% have reported problematic smartphone use [12]. It has been demonstrated that excessive or problematic smartphone use is related to a variety of adverse mental health outcomes, such as increased symptoms of depression, stress and anxiety, and also lower self-esteem [13,14]. Furthermore, users who show problematic smartphone use generally report emotional avoidance or impulsive behaviours [15,16].

Although there has been a great deal of research on smartphone overuse and the risk of psychological consequences, there are still numerous gaps in the knowledge on the risk factors that contribute to this specific problematic behaviour [6]. Previous studies have examined different factors that may contribute to problematic smartphone use and addiction, such as socio-demographic characteristics (e.g., gender, age, profession and education level [17,18]), personality and psychological traits [1,19], psychological symptoms and disorders [14] and emotion dysregulation [20].

Regarding emotion regulation, the theory has been proposed that individuals who exhibit higher deficits in emotion regulation are more likely to engage in risky behaviours in an attempt to reduce or alleviate the experience of negative emotions [21]. Several authors have suggested that smartphones may be used to manage negative emotions [14,22], specifically for adolescents [4]. Drawing on the theory of the regulation of emotions [23], it is proposed that problematic smartphone users may deal with negative emotion by using their smartphones, as a maladaptive coping strategy related to avoidance and the inhibition of their negative emotion, instead of using adaptive coping strategies such as social support or problem-solving [20]. For example, Kim, Seo and David [24] found that individuals with depressive symptoms use their smartphones as a coping strategy to deal with the negative effects of their depression. Moreover, Elhai, Levine, Dvorak and Hall [20] indicated that the relationship between problematic smartphone use and anxiety symptoms was mediated by maladaptive emotion regulation strategies.

Adolescence is a development stage that is vulnerable to problematic smartphone usage because of the presence of stressful events and encounters that, together with poor emotion regulation skills, may lead adolescents to relieve their negative emotions by using smartphones in an excessive way [25,26]. Although adolescents with deficits in emotion regulation are prone to the development of problematic use, it has also been shown that not all of them will become problematic users and, in turn, not all of them will develop adverse psychological symptoms and/or reduced well-being [18]. Taken together, it seems fruitful to investigate which specific difficulties in emotion regulation make adolescents susceptible to engaging in problematic smartphone use.

As adolescents may engage in excessive and addictive smartphone behaviour in reaction to negative emotional states, researchers have begun to analyse the affective factors that may play a role in smartphone overuse and problematic behaviours. It is argued that, during adolescence, emotion regulation becomes a key cognitive process to effectively manage mood states after experiencing stressful events or negative emotions [27,28]. Substantial evidence has also shown that the use of adaptive emotion regulation strategies is typically a key factor for preventing negative risky behaviours [25]. Garnefski and Kraaij’s [29] emotion regulation model proposes that there are relevant individual differences in how people can regulate the impact of negative emotions by using adaptive and maladaptive cognitive emotion strategies. Cognitive emotion regulation (CER) is described as the “conscious, mental strategies individuals use to cope with the intake of emotionally arousing information” [30], and it involves four maladaptive and five adaptive strategies. The four maladaptive CER strategies are rumination, self-blame, blaming of others and catastrophising, and they can lead to psychological and emotional problems such as depression, anxiety or risky behaviours [27,30]. By contrast, positive refocusing, refocusing on planning, acceptance, putting into perspective and positive reappraisal are the five adaptive strategies that are related to better mental health and well-being [27,31]. Specifically, a number of previous studies have linked the absence of adaptive strategies and the presence of maladaptive strategies with psychological adjustments, such as anxious and depressive symptoms, anger and distress [29,32] and different forms of psychopathology [33]. For instance, adolescents who reported lower use of all strategies and those who reported frequent use of the maladaptive strategies showed more psychopathological behaviours compared to those who reported average use of all strategies [28,34]. Additionally, individuals who use maladaptive CER strategies are more likely to exacerbate their engagement in risky behaviour, such as excessive alcohol use [35] and clinical drug addiction [36]. Thus, gaining knowledge about how maladaptive emotion regulation strategies are related to the development of problematic smartphone use is an important part of measuring and developing more effective prevention and intervention programmes.

In order to assess cognitive coping as one of the many ways to manage emotions associated with life stressors, the Cognitive Emotion Regulation Questionnaire (CERQ [27]) was developed. This instrument was designed to focus on all cognitive coping strategies at the same time in order to obtain a more complete and useful measurement compared to the assessment of a single coping strategy [27]. It includes the nine conceptual scales on rational bases that refer to what you think and actually do after experiencing a stressful situation [27]. By distinguishing between the specific CER strategies followed by adolescents with problematic use of smartphones, a better understanding of self-regulatory, conscious and cognitive processes of emotion regulation involved in smartphone overuse can be achieved and this can lead to more targeted and tailored strategies to prevent smartphone overuse.

Consequently, this study attempts to analyse the links between the use of specific CER strategies and problematic smartphone use in a sample of adolescents. First, we sought to examine whether certain CER strategies are applied differently by problematic and non-problematic smartphone users. Then, according to the theoretical framework of CER proposed by Garnefski et al. [27] and prior empirical evidence showing that maladaptive strategies are more consistently related to psychopathology than adaptive strategies [32], we hypothesized that: (H1) Emotion regulation strategies would be associated with problematic smartphone use behaviour; (H2) problematic smartphone users would report higher scores on maladaptive strategies and lower scores on adaptive strategies than non-problematic users; and (H3) maladaptive strategies would be best able to distinguish between problematic and non-problematic smartphone users.

## 2. Materials and Methods

### 2.1. Participants

The participants were 845 students (390 males and 455 females) from different secondary schools in southern Spain. The age of the adolescents ranged from 14 to 18 years, with a mean age of 15.64 years (SD = 1.16). The school years of the students involved in the study were the third year of E.S.O. (compulsory secondary education) to the second year of Bachillerato (high school grades 9–12). Furthermore, 95.8% of the participants were identified as Spanish.

### 2.2. Measures

The participants completed a package survey that included three sections: (1) Socio-demographic variables, (2) smartphone usage patterns and (3) the Spanish versions of the CERQ and SAS-SV (see below).

The socio-demographic variables measured were: gender, age and education level (i.e., from grades 9–12). The variables related to the patterns of smartphone use included: Parental control of their smartphone use at home; parental control of their smartphone use outside the home; average number of hours per weekend spent on their smartphone; average number of hours per week spent on their smartphone; whether or not they possessed their own smartphone.

The Cognitive Emotion Regulation Questionnaire (CERQ [27]) is composed of 36 items that assess a person’s CER strategies in response to threatening or stressful life events. The CERQ contains nine distinct subscales: five subscales for adaptive strategies (acceptance, positive refocusing, refocusing on planning, positive reappraisal and putting into perspective) and four subscales for maladaptive strategies (self-blame, rumination, catastrophising and blaming others) [29]. Four items are used to evaluate each of the CER strategies, measured on a five-point scale from 1 (“Almost never”) to 5 (“Almost always”) in terms of frequency of use. The Spanish CERQ has good levels of reliability and validity [37]. In the current research, the internal consistency for the different subscales ranged from 0.65 to 0.82.

The short version of the Smartphone Addiction Scale (SAS-SV [8]) is a validated scale developed from the original Smartphone Addiction Scale (SAS [38]). The SAS-SV is a unidimensional scale that contains ten items answered using a six-point Likert scale from 1 (“Strongly disagree”) to 6 (“Strongly agree”). The total score ranges from 10 to 60, with higher scores on the SAS-SV reflecting higher problematic use of a smartphone in the past year. We used the well-validated Spanish version of SAS-SV [8]. In this study, Cronbach’s alpha reliability coefficient was 0.85.

### 2.3. Procedure

All procedures performed in this study were in accordance with the ethical standards of the Research Ethics Committee of the University of Malaga (approval number 62-2016-H) and with the 2013 Declaration of Helsinki. Written consent for the individual participants was provided by the school authorities, who were responsible for consulting with and reporting to the parents about the research and who made the final decision on participation in the research. The assessment was therefore carried out in classrooms during the normal school day, with guarantees given that the participants were not obliged to participate and that their replies were anonymous, and with the written approval of the school authorities. Questionnaires were completed in a typical 1-hour lesson during the last two trimesters of the 2017/2018 academic year. The questionnaires were administered to the classes in sessions at which one of the researchers and at least one teacher from the school were present. All the participants were encouraged to provide honest answers.

### 2.4. Data Analysis

Statistical analyses were carried out using the Statistical Package for the Social Sciences [39] to calculate means, standard deviations and reliabilities for the measured variables, correlation coefficients, a multivariate analysis of variance (MANOVA) and a logistic regression analysis. To compare which of the emotion regulation strategies was associated with problematic smartphone use behaviours, the scores of the regulation strategies for participants with problematic and non-problematic smartphone use were compared. Using the cut-off points from Lopez-Fernandez [8] with a Spanish sample, the cut-off value of 32 was used for dividing the sample of adolescents into two groups: Problematic and non-problematic smartphone users. MANOVA was carried out to determine differences in CER strategies between the high and low SAS-SV scorers. Gender, age, educational level and parental control at home and outside were considered as covariates. *F*-tests were used to study the bivariate differences, and a significant main effect was identified.

Logistic regression analysis was performed to study the differences between smartphone users with regard to the nine CER strategies. In the present analysis, the binary dependent variable referred to membership of the high and low SAS-SV score groups (problematic and non-problematic smartphone users, respectively), whereas the CER strategies were defined as the independent variable set. To detect any multicollinearity between the nine CER strategies, we used the tolerance and a variance inflation factor (VIF), with tolerance below 0.10 and VIF greater than 10 considered to represent a multicollinearity problem. For all analyses, a significance threshold of *p* < 0.05 was established.

## 3. Results

### 3.1. Descriptive Analysis in Measured Demographic and Psychological Variables

The participants’ average age was 15.64 years, and the average age at which they started to use a smartphone was 11.65 years. Regarding the participants’ gender, 390 were boys (46.2%) and 455 were girls (53.8%). Their maximum education level was 12th grade (11.0%) and their minimum education level was 9th grade (45.7%). The participants’ parents controlled their smartphone use at home rarely (29.5%) or never (29.0%). At the same time, 40.0% of participants informed us that their parents never controlled their use of a smartphone outside the home. The average number of hours of smartphone use per week and per weekend was more than six hours for 59.8%and 57.4% of participants, respectively. A total of 92.8% of the participants had their own smartphone (see Table 1).

### 3.2. Differences in CER Strategies between Participants with High and Low SAS-SV Scores

The means and standard deviations in Table 2 for both excessive and non-excessive smartphone users show that refocusing on planning was the most commonly reported strategy. MANOVA showed a significant main effect on strategies for excessive and non-excessive smartphone users (Wilks’ *λ* = 0.944, *F* (9, 835) = 5.49, *p* < 0.001). Univariate *F*-tests showed significant differences between problematic and non-problematic smartphone users in relation to self-blame (*F* (1, 843) = 6.55, *p* = 0.011, *η*^2^ = 0.18), rumination (*F* (1, 843) = 23.49, *p* < 0.001, *η*^2^ = 0.34), catastrophising (*F* (1, 843) = 26.54, *p* < 0.001, *η*^2^ = 0.32) and blaming others (*F* (1, 843) = 11.18, *p* < 0.001, *η*^2^ = 0.23). Excessive smartphone users reported higher scores for these CER strategies than the non-excessive users.

The correlation between the CERQ subscales and smartphone addiction scores are presented in Table 3. Pearson correlation coefficients ranged from −0.01 to 0.63. In general, these results showed small to moderate correlations between the strategies.

### 3.3. Pearson Intercorrelation Coefficients Between the CERQ Subscales and the SAS-SV Scores

The correlations between the CERQ subscales and smartphone addiction scores are presented in Table 3. The Pearson correlation coefficients ranged from −0.01 to 0.63. In general, these results showed small to moderate correlations between the strategies.

### 3.4. Prediction of Problematic or Non-Problematic Smartphone User Group: Logistic Regression Analysis

A two-step logistic regression analysis was performed for excessive and non-excessive smartphone users (Table 4). In the first step, education level, gender, age and parental control of smartphone use at home and outside were considered as covariates. In the second step, the CER strategies were entered in the analysis as independent variables, yielding a significant model (*X*^2^_(5)_ = 43.30, *p* < 0.001) and explaining 9.3% of the variance (Cox and Snell *R*^2^). The tolerance index ranged from 0.37 to 0.97, while the VIF ranged from 1.03 to 2.64, indicating an absence of multicollinearity between the CER strategies included in the model. The Wald statistic was used to determine the significance of the contribution of the independent variables. A *β* logistic regression coefficient was used to determine the relative influence of the separate independent variables. Along with gender and parental control of the adolescent’s smartphone use, the results showed that three independent and maladaptive CER strategies (rumination, catastrophising and blaming others) were the most important variables for distinguishing between the two groups.

## 4. Discussion

While the advent of smartphone technology has led to numerous positive changes in all facets of human life, including business, education and health, several negative consequences associated with patterns of smartphone usage have increased alarmingly, especially in adolescents. These negative effects are linked to the impact of problematic smartphone usage on people’s health and well-being. As the problematic use of smartphones is becoming a risky behaviour in adolescents, with adverse academic and social consequences [26,40], an increased understanding of the specific psychological mechanisms associated with higher problematic use is likely to be useful in future intervention programmes. Among these psychological factors, the use of maladaptive coping strategies and potential deficits in emotion regulation are considered key factors that contribute to the excessive smartphone use in adolescents as a way of coping with stress and negative mood. Although past studies have clearly shown that deficits in emotion regulation skills are related to smartphone overuse, this is the first to distinguish between specific CER strategies followed by Spanish adolescents with problematic smartphone use.

As expected, our comparative analysis showed that problematic smartphone users reported significantly higher scores for maladaptive CER strategies, including higher self-blame, rumination, blaming of others and catastrophising, compared to non-problematic users. These findings are consistent with prior research that suggests that deficits in emotion regulation and the use of negative emotion regulation strategies might influence the problematic use of smartphones as a way of dealing with negative emotions [14,18]. Adolescence is a developmental stage in which individuals experience a plethora of stressful family, social and academic events and encounters [41]. Adolescents facing the typical psychosocial stressors may therefore rely on technologies such as mobile phones, computers, the internet or video games to reduce their negative feelings and overwhelming emotions [42,43]. Nevertheless, no group differences were found in any of the adaptive coping strategies. Based on our results, we tentatively propose that adolescents who are more likely to use ineffective coping strategies when they are in stressful situations might use smartphones excessively as a way of compensating for the negative feelings and thoughts associated with these maladaptive strategies. Our findings suggest that school counsellors might teach adolescents that stressful experiences have a cumulative negative impact on them, which might lead to problematic smartphone use as a form of negative regulation strategy. Also, it might be best for school intervention programmes aimed at the prevention of smartphone overuse to focus on how to identify negative events, how to recognize potential symptoms of stress and how to reduce negative coping patterns (i.e., rumination, catastrophising, etc.) so that adolescents are better able to manage negative emotions such as sadness, worry and anxiety, thus reducing the likelihood of subsequent mental health problems and technology abuse.

Additionally, our findings from the bivariate analyses suggest that individual differences in the propensity among adolescents to experience higher problematic smartphone use were associated with high self-blame, blaming of others, rumination and catastrophising, and also with low positive reappraisal. These findings give credibility to prior empirical evidence on deficits in emotional regulation and greater use of maladaptive coping strategies in technology addictions [44]. Accordingly, it is possible that adolescents using maladaptive coping strategies engage in higher problematic smartphone use to avoid or reduce their negative feelings but, alternatively, they might engage in the excessive use of technology as a way to prolong their positive mood states if they believe themselves to be incapable of using alternative ways to foster positive emotions [45]. A fruitful line of research would be to examine the potential psychological motives (i.e., reducing negative moods and/or increasing positive moods) that lead adolescents to use their smartphones excessively.

In addition, our research used binary logistic regression to examine, simultaneously, the extent to which CER strategies account for potential differences observed between the problematic and non-problematic user groups. In general, our results showed that gender, parental control outside the home and three maladaptive CER strategies (rumination, catastrophising and blaming others) are the main contributors to the prediction of belonging to the problematic rather than the non-problematic user group.

Regarding socio-demographic variables, gender appeared as a significant predictor. In line with past studies, female adolescents were more prone to experience dependence on their smartphones [46]. This association between the female gender and smartphone overuse might be partly related to the fact that females tend to value interpersonal communication more and typically show higher levels of social connectedness than males [47]. Similarly, prior studies have reported the protective effect of parental control and mediation on smartphone use [48]. Our findings are in line with this literature suggesting that parental control outside the home might be an effective strategy to address problematic smartphone use in adolescents. Therefore, further prevention and intervention programmes on mobile phone addictions might usefully target not only the adolescents but also the parents, encouraging them to engage in active parental control through modelling, monitoring and mediating, which might reduce the adolescent behavioural risk over smartphone use [43,49].

Finally, regarding maladaptive CER strategies, a higher reported use of the strategies of rumination, catastrophising and blaming others independently contributed to the prediction of belonging to the problematic rather than the non-problematic user group. That said, those adolescents who engaged in thinking repetitively about the feelings and thoughts associated with a negative event, emphasising the negative aspects of an event that they had experienced and usually putting the blame for what they had experienced on the environment or another person, reported significantly higher problematic use of their smartphones. This negative coping pattern associated with problematic smartphone use corroborates past work that indicated that rumination, catastrophising and blaming others were risk factors for developing mental health problems [27,33], but also expands these findings by suggesting that this set of maladaptive coping strategies is key in the prediction of excessive smartphone use. However, the use of these maladaptive CER strategies together accounted for a significant, although modest, amount of the variance, suggesting that other psychosocial, demographical and family factors (i.e., peer influence, duration of smartphone usage, etc.) also affect problematic smartphone behaviours. More integrated models that include these factors should be examined in further research. As a practical implication, given that researchers have pointed out that these coping strategies may be changed and learned through school programmes for adolescents [25], our data might serve as a good starting point when treating problematic smartphone users in adolescence with the inclusion of modules to identify profiles of CER deficits and as a complement to cognitive-behavioural interventions to reduce maladaptive strategies (e.g., rumination, catastrophizing and blaming others) [50]. Furthermore, it is noteworthy that once we had controlled for socio-demographic variables, parental control and the other cognitive strategies, the independent effects of positive reappraisal and self-blame found in the correlation analyses disappeared. In line with prior work [27], our results should not lead to the conclusion that the strategies of self-blame and positive reappraisal are unimportant in the overuse of technology, but these findings give some support to the performance of an integrated and unified analysis. A better understanding of the joint role of cognitive strategies in problematic smartphone use might not depend on one specific strategy but on a combined set of various coping strategies.

There are some limitations of our study that point to directions for future research. First, this study was cross-sectional, thus precluding causal interpretations. It is obvious that longitudinal research would provide more valid data on the causal relationship between CER strategies and the problematic use of a smartphone over time. Furthermore, the results rely on self-reported measures to evaluate emotion regulation strategies and problematic smartphone use. To address these concerns, the new clinical approaches (such as a diagnostic interview, clinician-administered instruments or expert judgements) or a new approach using a situational judgement test for measuring emotional abilities (such as performance-based methodologies) would provide a more comprehensive perspective and is an important avenue for research moving forward. Similarly, our results only refer to the conscious CER strategies that adolescents reported using in stressful situations; no other forms of coping, such as behavioural strategies or strategies focused on social support, were evaluated. Such complementary coping elements should be included in more comprehensive theoretical models to increase our understanding and assist with the further development of successful coping intervention programmes. Similarly, our study was based on a convenience sample of adolescents, and we did not have data on psychiatric diagnoses or the frequency of medical problems in our sample. Finally, our findings used a non-random design in a healthy population and, therefore, may not necessarily generalise to other populations.

Notwithstanding the above-mentioned limitations, our study provides a knowledge base on the potential deficits in coping strategies for technology addiction and suggests that it is important to pay attention to maladaptive strategies in the development of intervention programmes for smartphone overuse. Thus, according to a transdiagnostic approach, problematic use of the smartphone might be conceptualised within a broader spectrum of technological problematic use that encompasses a variety of dysfunctional behaviours and implies involvement in specific online activities. Deficits in emotion regulation and maladaptive coping profiles have been found in other compulsive technology use (i.e., internet, video games, gambling) [44]. These maladaptive coping strategies can be viewed as shared risk factors and might be an underlying and transdiagnostic process to a wide variety of technology addictions. It is tentative to think that adolescents with poorly regulated emotions often engage in maladaptive coping related to technology abuse to down-regulate their mood states, which involves negative consequences for mental health [51]. Expanding our findings, future work should examine whether a specific maladaptive coping profile (i.e., rumination, catastrophising) might be an affective transdiagnostic process related to different technological addictions. Such a process will be beneficial for prevention policies and comprehensive intervention programmes for affected adolescents.

Through cognitive–behavioural treatment, which is a widely used method for changing negative thoughts and behaviours, a therapist might modify certain maladaptive CER strategies, especially in adolescents who demonstrate this cognitive coping profile, and supply more adaptive strategies such as positive reappraisal [25]. Also, educational seminars based on maladaptive coping strategies or experiences of the adverse results of smartphone overuse and behaviours can be implemented, with the aim of teaching the appropriate use of smartphones by using a variety of evidence-based positive cognitive patterns [52,53]. Therapists and school counsellors can assist adolescents who have problematic smartphone use to recognise the thoughts and maladaptive coping (i.e., rumination, catastrophising and blaming others) that cause them to use their smartphone inappropriately to meet their personal needs [54]; this should be complementary to digital interventions for problematic smartphone use (i.e., apps that can help adolescents to track their smartphone use and set goals for moderating their use) [53]. Thus, our findings might be beneficial for school psychologists trying to identify high-risk adolescents, given that the typical use of maladaptive coping strategies can potentially be used as a screening assessment to identify students who are more likely to develop excessive technology use.

## 5. Conclusions

Although these findings warrant replication, our research emphasises the importance of research that examines how adolescents suffer from problematic smartphone use and the potential causes, linked with coping styles for reducing mood, contributing to a fruitful target for the prevention of and intervention in psychological impairment associated with technology addiction in adolescents.

## Figures and Tables

**Table 1:** Descriptive analysis in the socio-demographic variables and patterns of smartphone use.

Socio-Demographic Variables	Sample	
	M	SD
Age	15.63	1.16
Average starting age of mobile phone use	11.65	1.65
	*N*	%
Gender		
Male	390	46.2
Female	455	53.8
Educational level		
9th grade secondary	386	45.7
10th grade secondary	246	29.1
11th grade secondary	120	14.2
12th grade secondary	93	11.0
Parental control at home		
Never	245	29.0
Rarely	249	29.5
Occasionally	197	23.3
Often	113	13.4
Always	41	4.9
Parental control outside the home		
Never	338	40.0
Rarely	226	26.7
Occasionally	155	18.3
Often	78	9.2
Always	48	5.7
Hours of smartphone home use per week		
I do not use it	15	1.8
Less than 2 h	45	5.3
2 to 3 h	85	10.1
3 to 5 h	195	23.1
More than 6 h	505	59.8
Hours of smartphone use per weekend		
I do not use it	6	0.7
Less than 2 h	41	4.9
2 to 3 h	98	11.6
3 to 5 h	215	25.4
More than 6 h	485	57.4
To own a smartphone		
No, I do not use any	2	0.2
No, but use someone else’s	5	0.6
Yes, I have one of my own	784	92.8
Yes, I have more than one of my own	54	6.4

**Table 2:** Differences in Cognitive Emotion Regulation strategies between high and low Smartphone Addiction (SAS-SV) scores.

Cognitive Emotion Regulation Strategies	Low SAS-SV Scores (*N* = 491)		High SAS-SV Scores (*N* = 354)			
	M	SD	M	SD	*F*	*η* ^2^
Self-blame	11.10	3.29	11.70	3.34	6.55 *	0.18
Acceptance	13.32	3.44	13.70	3.16	2.64	0.11
Rumination	12.34	3.73	13.55	3.31	23.49 ***	0.34
Positive refocusing	11.30	4.31	11.10	4.15	0.47	0.04
Refocus on planning	14.26	3.82	13.97	3.80	1.17	0.07
Positive reappraisal	13.72	3.97	13.34	3.86	1.92	0.09
Putting into perspective	13.68	3.86	13.55	3.47	0.24	0.03
Catastrophising	9.48	3.26	10.66	3.31	26.54 ***	0.32
Blaming others	8.52	3.21	9.26	3.12	11.18 ***	0.23

*η*^2^ = partial eta-squared. * *p* < 0.05; *** *p* < 0.001.

**Table 3:** Pearson intercorrelations between Cognitive Emotion Regulation strategies subscales and excessive smartphone scale.

Variables	1	2	3	4	5	6	7	8	9
1. Self-blame	--								
2. Acceptance	0.43 **	--							
3. Rumination	0.50 **	0.50 **	--						
4. Positive refocusing	0.05	0.25 **	0.15 **	--					
5. Refocus on planning	0.31 **	0.43 **	0.41 **	0.43 **	--				
6. Positive reappraisal	0.18 **	0.37 **	0.21 **	0.48 **	0.63 **	--			
7. Putting into perspective	0.20 **	0.34 **	0.23 **	0.38 **	0.51 **	0.60 **	--		
8. Catastrophizing	0.34 **	0.27 **	0.42 **	0.06	0.05	−0.01	0.10 **	--	
9. Blaming others	−0.17	0.08 *	0.15 **	0.17 **	0.11 **	0.09 *	0.16 **	0.34 **	--
10. Smartphone Use	0.13 **	0.06	0.23 **	−0.02	−0.05	−0.08 *	−0.03	0.21 **	0.15 **

* *p* < 0.05, ** *p* < 0.01.

**Table 4:** Identification of CER strategies to distinguish between problematic and non-problematic smartphone users.

Variables	*β*	SE*β*	Wald	*p*	VIF	Tolerance
Gender	0.73	0.15	21.86	*p* < 0.01	1.03	0.97
Age	0.18	0.10	3.11	*p* = 0.07	2.64	0.37
Educational level	−0.06	0.11	0.32	*p* = 0.57	2.61	0.38
Parental control at home	0.07	0.07	0.87	*p* = 0.34	1.51	0.66
Parental control outside	−0.23	0.07	9.04	*p* < 0.01	1.53	0.65
Self-blame	0.02	0.02	0.65	*p* = 0.42	1.56	0.64
Acceptance	−0.01	0.02	0.02	*p* = 0.86	1.63	0.61
Rumination	0.06	0.02	5.33	*p* = 0.02	1.93	0.51
Positive refocusing	0.01	0.02	0.34	*p* = 0.55	1.44	0.69
Refocus on planning	−0.05	0.02	3.16	*p* = 0.07	2.16	0.46
Positive reappraisal	−0.01	0.02	0.07	*p* = 0.78	2.27	0.43
Putting into perspective	−0.02	0.02	0.69	*p* = 0.40	1.73	0.57
Catastrophizing	−0.05	0.02	4.25	*p* = 0.03	1.51	0.66
Blaming others	0.06	0.02	5.96	*p* = 0.01	1.24	0.80

Total explained variance (Cox and Snell *R*^2^): 9.3%. Significance model: *X*^2^_(5)_ = 43.30, *p* < 0.001. Abbreviation: SE*β*: Standard error.
